# 

**Published:** 1906-03-31

**Authors:** 


### NOTICE TO CORRESPONDENTS.

All MSS., Letters, Books for Review, and other matters intended for the Editor should be addressed The Editor, "The Hospital," The Hospital Buildings, 28 & 29 Southampton Street, Strand, W.C.

The Editor cannot undertake to return rejected MS., even when accompanied by stamped directed envelope.

**Notice to Advertisers and Subscribers.**

All Advertisements, Orders for Copies of The Hospital, and Business Communications should be addressed to **THE MANAGER (Not to the Editor),** The Hospital, 28 & 29 Southampton Street, Strand London, W.C.

The Cost of Subscription, post free, is as follows:—Per week, 3½d.; per quarter, 3s. 3d.; per half-year, 6s. 6d.; per annum, 13s. Foreign and Colonial:—Per annum, 19s. 6d., payable, in all cases, in advance.

**NOTICE.—Vol. XXXVIII. of "The Hospital" April to September 1905), in handsome cloth binding, will shortly be on sale at the office of this Journal, price 10s. 6d. Cases for binding the Numbers contained in this Volume 2s. Index to Vol. XXXVIII., 3½d., post free.**

**The Monthly Part for March is now ready. Price 1s., by post, 1s. 3d.**

### BOOKS RECEIVED.

Baillière, Tindall, and Cox.

"Aids to Surgical Diagnosis." By H. W. Carson, F.R.C.S.

W. Heinemann.

"Sex and Character." By Otto Weininger.

The Scientific Press, Limited.

"Burdett's Hospitals and Charities, 1906."

J. Wright and Co.

"First Aid to the Injured and Sick." By Warwick and Tunstall. Fourth edition.

Potter and Clarke.

"Potter's Cyclopædia of Botanical Drugs and Preparations."

J. Bale, Sons and Danielsson, Limited.

"On Means for the Prolongation of Life." By Sir Hermann Weber, M.D.

W. H. and L. Collingridge.

"City of London Directory, 1906."

P. Blakiston's, Sons and Company.

"The World's Anatomists." By G. W. H. Kemper, M.D.

The Author.

"The Causation of Cancer." By M. L. Johnson.

J. B. Lippincott Co.

"International Clinics." Fifteenth Series. Vol. IV.

A. H. Stockwell.

"Peeps in the Past." By F. E. Tyter.

### MEDICAL APPOINTMENTS AND VACANCIES.

### APPOINTMENTS.

Tylecote, Frank E., M.D., D.P.H., C.H.B., Senior Assistant Medical Officer to the County Borough of West Ham Fever Hospitals, Plaistow and Dagenham.

### VACANCIES.

Birkenhead Borough Hospital.—Junior Resident House Surgeon.

Bournemouth, Royal Boscombe, and West Hants Hospital.—House Surgeon.

Bridgwater Hospital.—House Surgeon.

Bristol Eye Hospital.—House Surgeon.

Bury Infirmary.—Junior House Surgeon.

Charing Cross Hospital.—Resident Medical Officer.

City of London Hospital for Diseases of the Chest, Victoria Park, E.—Two House Physicians.

Durham County Hospital.—House Surgeon.

East London Hospital for Children and Dispensary for Women, Shadwell, E.—Medical Officer for the Casualty Department.

Egyptian Government, Kasr-el-Ainy Hospital.—Resident Surgical Officer.

Hospital for Consumption and Diseases of the Chest, Brompton.—Resident House Physicians.

Huddersfield Infirmary.—Senior House Surgeon.

Leeds Public Dispensary.—Junior Resident Medical Officer.

Lincoln County Hospital.—Junior House Surgeon.

Oldham Infirmary.—Senior House Surgeon.

Perthshire, Dunblane Hydropathic.—Physician.

Portsmouth, Royal Portsmouth Hospital. — House Physician.

Public Dispensary, Drury Lane, W.C.—Physician.

Queen Charlotte's Lying-in Hospital, Marylebone Road, N.W.—Pathologist and Registrar.

Royal Hospital for Diseases of the Chest, City Road, E.C.—Clinical Assistants.

Samaritan Free Hospital for Women, Marylebone Road, N.W.—Anæthetist. Also Clinical Assistants.

Seamen's Hospital Society, Greenwich, S.E.—Dreadnought Hospital.—Two House Physicians and Two House Surgeons. Branch Hospital, Royal Albert Dock, E.—Senior House Surgeon. Also House Surgeon.

Swansea General and Eye Hospital.—Assistant House Surgeon.

Wakefield, Clayton Hospital and Wakefield General Dispensary.—Junior House Surgeon.

Wrexham Rural District Council, Denbigh.—Medical Officer of Health and Superintendent of the Joint Fever Hospital.

### HANDBOOKS FOR MOTHERS AND NURSES

### The Mother's Help.

**A GUIDE TO THE DOMESTIC MANAGEMENT OF CHILDREN IN HEALTH AND DISEASE.**

By P. MURRAY BRAIDWOOD, M.D.

Second Edition, bound in cloth, **2/-** post free.

CONTENTS: The Signs of Health—Management of the Newly-Born—Great Mortality during Infancy and Childhood—The Feeding of Children—On Children's Constitutions—On Clothing Children—On Children's Exercises and Amusements—The Domestic Management of Children When Ill—On Infant Feeding—Education.

**Provincial Medical Journal** says: "To young mothers it should prove invaluable."

### The Nursing of Sick Children.

By JAMES BURNET, M.A., M.D., M.R.C.P.Edin.

Bound in cloth, **1/-** net; **1/1** post free.

CONTENTS: The Feeding of Sick Children—Baths: When and How to Give Them—On the Management of Respiratory Cases, Vomiting and Diarrhœa Cases—On the Management of Rheumatic Cases—Diseases of the Nervous System—Paralysed and Spinal Cases—Rickets and Scorbutus—Skin Cases—Diseases of the Eye, Ear, Nose and Throat.

### Sight and Hearing in Childhood.

By ROBT. BRUDENELL CARTER, F.R.C.S., and ARTHUR H. CHEATLE, F.R.C.S.

Illustrated, bound in cloth, **2/-** net; **2/3** post free.

### The Art of Feeding the Invalid.

A Series of Chapters on the Nature of certain Prevalent Diseases and Maladies, together with carefully selected Recipes for the Preparation of Food for Invalids.

By A MEDICAL PRACTITIONER and A LADY PROFESSOR OF COOKERY.

New Edition, with an important new Chapter on Food in Tuberculosis and Consumption of the Lungs.

Bound in cloth, **1/6** post free.

### Diet in Sickness and in Health.

By MRS. ERNEST HART.

A most useful book to those who are sick, and to those who have to nurse, feed and prescribe for the sick, and it should be of great assistance to the healthy to preserve health.

Profusely illustrated, bound in cloth, **3/6** post free.

### Spinal Curvature and Awkward Deportment:

**THEIR CAUSES AND PREVENTION IN CHILDREN.**

By DR. GEORGE MÜLLER, Professor of Medicine and Orthopædics, Berlin.

English Edition, Edited and Adapted by RICHARD GREENE, F.R.C.P.Ed.

Bound in cloth, with Plates and Illustrations, **2/6** post free.

***Complete Catalogue post free on application.***

**The Scientific Press, Ltd.,** 28 & 29 SOUTHAMPTON STREET, STRAND, LONDON, W.C.

**Publishers and Booksellers,**

